# Gamma Radiation Effects on Peanut Skin Antioxidants

**DOI:** 10.3390/ijms13033073

**Published:** 2012-03-07

**Authors:** Adriano Costa de Camargo, Thais Maria Ferreira de Souza Vieira, Marisa Aparecida Bismara Regitano-D’Arce, Maria Antonia Calori-Domingues, Solange Guidolin Canniatti-Brazaca

**Affiliations:** 1 Center for Nuclear Energy in Agriculture, University of São Paulo (CENA/USP), Av. Centenário 303, P.O. Box 96, 13400-970, Piracicaba, SP, Brazil; 2 Department of Agri-Food Industry, Food & Nutrition, “Luiz de Queiroz” College of Agriculture (ESALQ/USP), University of São Paulo, Av. Pádua Dias 11, P.O. Box 9, CEP 13418-900, Piracicaba, SP, Brazil; E-Mails: tvieira@usp.br (T.M.F.S.V.); marisadarce@usp.br (M.A.B.R.-D.); macdomin@esalq.usp.br (M.A.C.-D.); sgcbraza@usp.br (S.G.C.-B.)

**Keywords:** peanut skin, residue, antioxidant activity, soybean oil, lipid oxidation

## Abstract

Peanut skin, which is removed in the peanut blanching process, is rich in bioactive compounds with antioxidant properties. The aims of this study were to measure bioactive compounds in peanut skins and evaluate the effect of gamma radiation on their antioxidant activity. Peanut skin samples were treated with 0.0, 5.0, 7.5, or 10.0 kGy gamma rays. Total phenolics, condensed tannins, total flavonoids, and antioxidant activity were evaluated. Extracts obtained from the peanut skins were added to refined-bleached-deodorized (RBD) soybean oil. The oxidative stability of the oil samples was determined using the Oil Stability Index method and compared to a control and synthetic antioxidants (100 mg/kg BHT and 200 mg/kg TBHQ). Gamma radiation changed total phenolic content, total condensed tannins, total flavonoid content, and the antioxidant activity. All extracts, gamma irradiated or not, presented increasing induction period (h), measured by the Oil Stability Index method, when compared with the control. Antioxidant activity of the peanut skins was higher than BHT. The present study confirmed that gamma radiation did not affect the peanut skin extracts’ antioxidative properties when added to soybean oil.

## 1. Introduction

Peanut skins are waste from the blanching process of peanuts, which are recognized for their bioactive compounds’ antioxidative properties. Although these skins are a potential source for natural antioxidants, they have only been used for animal feed. Proanthocyanidins [1] and flavonoids [2] have been isolated from the water-soluble fraction of peanut skins. Radical scavenging activity in relation to the content of total phenolic compounds and trans-resveratrol in fractions from ethanolic extracts of peanut skins was reported [3]. Proanthocyanidins isolated from the water-soluble fraction of peanut skins exhibited antioxidant activity [4]. The optimization of the extraction conditions for the recovery of antioxidant compounds from peanut skins was reported and ethanol was the most efficient solvent for peanut skin extraction, followed by methanol and water [5]. A study [6] found that phenolic compounds extracted from peanut skins could significantly reduce the oxidation of meat products and extend their storage stability. While the blanching process influences both the total phenolic and isolated procyanidin contents [7], the total phenolics and procyanidins present in skins that were peeled by hand or by roasting presented concentrations comparable to those found in grape seeds. In addition, the total antioxidant activity and free radical scavenging capacities of peanut skin extracts were all higher than those of Trolox or Vitamin C at equivalent concentrations.

Proanthocyanidin dimers and trimers from peanut skins can interact with membrane phospholipids, presumably with their polar headgroup. As a consequence of this interaction, they can provide protection against the attack of oxidants and other molecules that challenge the bilayer’s integrity [8]. Phenolic compounds extracted from defatted peanut skins presented a greater protective effect against the hemolysis of red blood cells than ascorbic acid under *in vitro* conditions [9].

Some studies have shown increased antioxidant activity in gamma irradiated products [10–14]. Furthermore, peanut skins samples are susceptible to microbial contamination which can be reduced with gamma radiation. This is already done successfully with spices. The objective of this study was to evaluate the effect of gamma radiation on peanut skins from the blanching process, with a focus on bioactive compounds and antioxidant properties. Total phenolics, total flavonoids, condensed tannins, and *in vitro* antioxidant activity were determined in the peanut skins. Additionally, the antioxidant activity of the peanut skin extract in soybean oil, a model system for the Oil Stability Index, was measured using Rancimat.

## 2. Results and Discussion

### 2.1. Polyphenols and Antioxidant Activity

Total phenolic content, condensed tannin, and total flavonoid content of peanut skin extracts are shown in Table 1. It has been noted in the literature that the amount of polyphenols is different for each peanut product. In gallic acid equivalents, peanut skins presented total phenolic content ranging from 97.0 [9] to 143.6 [15]. These values are in agreement with the present study. With regards to the irradiation process, a noticeable increase in total phenolic content at 7.5 and 10.0 kGy was observed. Also a positive correlation was found between gamma radiation doses and total phenolic content (*r* = 0.6905, *p* < 0.05). Two of the most common monofloral Malaysian honeys that were gamma irradiated with 25.0 kGy exhibited a two- or three-fold increase in their total phenolic contents [16]. Even doses as low as 2.0 kGy were able to increase the total phenolic content in peaches [17]. However, no significant effect was observed in the total phenolic content of radiation-processed tea leaves (0.0, 1.0, 2.0, 5.0, and 10.0 kGy) [18]. Similar results were noticed in the phenolic compounds of the green tea leaf and its byproduct after a 20.0 kGy irradiation [19]. Any differences among herbs gamma irradiated at 5.0, 10.0, or 15.0 kGy were found in terms of total phenolics and total flavonoids [20]. However, irradiation of almond skins with [11] doses of 4.0, 8.0, or 12.0 kGy increased total phenolics in samples purchased from Blue Diamond Growers and, likewise, doses of 12.7 or 16.3 kGy irradiated on samples purchased from Campos Brothers.

The most phenolic antioxidants present in lyophilized peanut skin extracts were condensed tannins (a total of 25.19% in peanut skins) [21], which agree with the present study. According to Tukey’s multiple test (*p* < 0.05) condensed tannin content was decreased by gamma radiation (Table 1). This decrease could be due to the degradation of tannins and could explain the higher total phenolic contents in the extracts [22]. Furthermore, 65.0 mg/g in catechin equivalents was reported for total flavonoid content [9]. In the present study, there was a slight increase in total flavonoids at 7.5 kGy. An increase in total flavonoid content was also reported for honeys that were gamma irradiated with 25.0 kGy [16]. However, differently of total phenolic content, no correlation was found among gamma radiation doses and condensed tannin (*r* = −0.3949) or gamma radiation doses and total flavonoid content (*r* = 0.5973), by Pearson’s coefficient.

**Figure 1:**
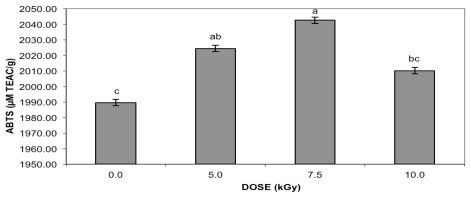
Antioxidant activity of gamma-irradiated peanut skin extracts measured by DPPH assay. Data represent the mean ± standard deviation of each sample assayed in triplicate. Means with different letters indicate significant differences among doses by Tukey’s multiple test (*p* < 0.05).

**Figure 2:**
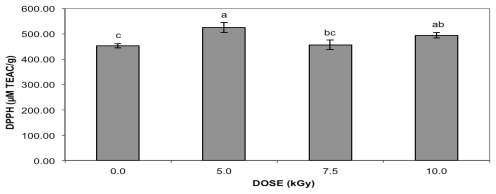
Antioxidant activity of gamma-irradiated peanut skin extracts measured by ABTS assay. Data represent the mean ± standard deviation of each sample assayed in triplicate. Means with different letters indicate significant differences among doses by Tukey’s multiple test (*p* < 0.05).

The DPPH assay showed increasing antioxidant activity at 5.0 and 10.0 kGy (Figure 1), while for ABTS (Figure 2), the effect was noticeable at 5.0 and 7.5 kGy. Similarly to condensed tannin and total flavonoid content, no correlation was found between antioxidant activity (DPPH, *r* = 0.3011 or ABTS, *r* = 0.5218) and gamma radiation doses. In addition to the irradiation process, the extraction and the assay method can affect the measured total phenolic content and the antioxidant activity of the peanut skin extracts. Values of 2149 and 2789 μmol TEAC/g were reported for the antioxidant activity of peanut skin extracts employing Oxygen Radical Absorbance Capacity (ORAC) using solid-liquid extraction (SLE) and microwave-assisted extraction (MAE), respectively [15]. The peanut type is another factor that influences the TEAC value. Studies with 27 peanut cultivars reported that the total antioxidant capacities from the peanut skins, measured by ABTS, ranged from 59.1 to 103.8 mM TEAC/100 g, with a mean value of 82.3 mM TE/100 g [23]. Different from the present study there was no significant difference in the DPPH radical scavenging capacity between irradiated (20.0 kGy) and control green tea leaves and byproducts [19].

Similarly to the present study, gamma-irradiated pomegranate peel [10] presented a slight increase in antioxidant activity, measured by ABTS, at 10.0 kGy. Higher scavenging activities of non-irradiated and irradiated ethanolic extracts of peanut skins and hulls when compared to TBHQ at 50 μg/mL using the DPPH radical was reported [24]. At this concentration, the gamma radiation did not change the scavenging activity of the samples. The antioxidant properties of tea [18], such as free radical scavenging activity measured by the DPPH assay, were not affected by a radiation treatment with a dose of 10.0 kGy. Gamma-irradiated almond skins [11] presented the most effective antioxidant activity by reducing the absorbance of ABTS radical at 4.0 and 12.0 kGy for samples purchased from different suppliers. Nonsignificant increases in the antioxidative capacity of cumin seeds irradiated with 1.0, 3.0, 5.0, or 10.0 kGy were found [25].

Dry rosemary leaf powder subjected to 30.0 kGy of gamma radiation followed by solvent extraction with methanol, ethanol or water presented EC_50_ values for DPPH assay that decreased by 22% for ethanol and water extracts compared to the control samples. EC_50_ values in the reducing power test showed a decrease of 45% and 28% for the ethanol and water extracts, respectively. No significant difference was observed for the methanolic extracts [12].

The increase in phenolic compounds by gamma radiation could be attributed to their release from glycosidic components and the degradation of larger phenolic compounds into smaller ones by gamma irradiation [11]. The increase in isoflavones in the aglycone form and decrease in glicosidic forms were also reported as a consequence of gamma radiation [26]. In the present study, gamma radiation could have affected the extraction efficiency during the analysis and the types of compounds extracted, increasing the total phenolic content and the antioxidant activities measured by the DPPH and ABTS assays. However, to confirm this hypothesis, other parameters such as the gamma radiation induced generation of Maillard compounds [27,28], which have demonstrated antioxidant properties should be eliminated. In addition, the identification and quantification of individual phenolic compounds need to be carried out. Both of the results of the present study and results from other researchers make it possible to say that gamma radiation does not negatively affect the free radical scavenging activity.

### 2.2. Analysis of the Peanut Skin Extracts Concentrations Using the Oil Stability Index Method

**Figure 3:**
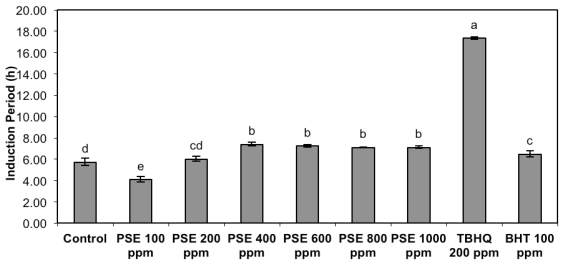
Induction period (h) of soybean oil with added peanut skin extracts (PSE). Data represent the mean ± standard deviation of each sample assayed in triplicate. Means with different letters indicate significant differences among doses by Tukey’s multiple test (*p* < 0.05).

The induction period from soybean oil with added peanut skin extracts is shown in Figure 3. At 100 mg/kg peanut skin extracts presented a pro-oxidant effect. This could be due to the extraction of pro-oxidant compounds that were more active than the antioxidants extracted at this concentration. Green tea extracts also presented pro-oxidant effect in marine oil. After dechlorophyllization the extracts presented antioxidant activity [29]. These results demonstrate that there is a competition between the pro-oxidant and antioxidant compounds that can be extracted. Pro-oxidant effect was also reported for α-tocopherol that is also known for its antioxidant properties. This behavior depends on the concentration of α-tocopherol as well as the solvent system. In an aqueous system, the pro-oxidant effect occurs more easily [30].

Peanut skin extracts showed the highest induction period at 400 mg/kg or more (Figure 3) once nonsignificant differences were noted from 400 to 1000 mg/kg. Although the induction periods of the extracts from 400 to 1000 mg/kg demonstrated lower values than those of TBHQ, antioxidant activity exists for these concentrations when compared to the control and BHT. Canola meal extracts [31] at 100, 200, 500 and 1000 ppm were able to reduce the peroxide value of canola oil submitted to accelerated oxidation. However, the reduction was not proportional to the concentration of the extracts, which was also noticed at the present study.

Vegetable oils used in several studies expressed TBHQ as the most effective antioxidant activity, even under high temperatures. However, the natural antioxidants from oregano extracts, rosemary, and sesame that were added to soybean oil demonstrated higher antioxidant effects than BHA or BHT [32]. Effectiveness of dechlorophyllized green tea extracts at 500 and 1000 ppm levels was superior to that of α-tocopherol at 500 ppm and BHA and BHT at 200 ppm, but somewhat less than that of TBHQ at 200 ppm [29], which agrees with the present study that expressed TBHQ as the most effective. Phenolic compounds extracted from peanut skins also reduced the oxidation from meat products and increased its shelf life [6].

### 2.3. Antioxidant Effect of the Irradiated Peanut Skin Extracts Using the Oil Stability Index Method

The induction periods of the soybean oil with added ethanolic extracts of gamma-irradiated peanut skins are shown in Table 2.

Gamma radiation did not affect the induction period of soybean oil added to ethanolic peanut skin extracts. Although, according to Tukey’s multiple test (*p* < 0.05), the antioxidant activities (DPPH and ABTS) increased with irradiation, the results were not observed during the Oil Stability Index assay. In fact, according to Pearson’s coefficient, no correlation was found between gamma radiation and antioxidant activity (DPPH or ABTS). It is possible to conclude that gamma radiation does not affect the antioxidant property of the peanut skin extracts and, in turn, the oxidative protection of soybean oil. Gamma-irradiated peanut skins showed longer induction periods than BHT and the control samples. However, they presented lower induction periods than TBHQ. These results agree with the findings of studies [11] that reported that the antioxidant properties of almond skin extracts added in soybean oil submitted to oxidation initiated with ferric chloride were not affected by gamma radiation at 2.8, 4.8, 8.8, 12.7, or 16.3 kGy when analyzed by peroxide value.

The lipid peroxidation of fish oil emulsion, measured by the presence of thiobarbituric acid reactive substances (TBARS), was inhibited by approximately 76%, while the superoxide radicals were scavenged by approximately 72% to 75% in the presence of tea leaves infusion *in vitro*. Gamma radiation doses of 1.0, 2.0, 5.0, or 10.0 kGy did not affect the antioxidant properties of the extracts prepared from the tea leaves [18].

Green tea leaf extract [33] decreased the oxidation in raw and cooked pork patties stored for 15 days under 4 °C. The TBARS value of the raw pork patties increased during the 15 days of storage in all treatments. However, the TBARS values of the patties with added freeze-dried green tea leaf extract powder (0.1%) were significantly lower than those of the control. There was no significant difference between irradiation with 20.0 kGy and non-irradiated extracts.

## 3. Experimental Section

### 3.1. Material

The peanut skin samples from cultivar IAC Runner 886 (crop year 2009/2010) were obtained from CAP—Agroindustrial, Dumont, São Paulo State, Brazil. The samples were separated in polyethylene plastic bags and irradiated with 5.0, 7.5, or 10.0 kGy at a dose rate of 7.5 kGy/h. One package was not irradiated for use as a control. Irradiation process was carried out in the city of São Paulo, São Paulo State, Brazil, using a multipurpose Cobalt-60 γ-irradiation facility from Nuclear Energy Research Institute (IPEN). IPEN is an autarchy, associated to the University of São Paulo—supported and operated technically and administratively by the National Nuclear Energy Commission (CNEN).

### 3.2. Methods

#### 3.2.1. Total Phenolics

Total phenolic content was analyzed in methanolic extracts with a concentration of 0.40 mg/mL, according to the Folin-Ciocalteu method [34]. First, 0.02 g of peanut skins in 50.00 mL of methanol was shaken for 20 min at room temperature. Second, the extract was centrifuged at 700 rpm for 15 min, and the supernatant was collected and transferred to a volumetric flask. Third, the volume was made up to 50.00 mL, and it was used as the final extract. Fourth, the extract (0.50 mL), deionized water (4.0 mL), and Folin-Ciocalteu reagent (0.50 mL) were added into flasks and mixed thoroughly. After 3 min, 1.00 mL of a Na_2_CO_3_ saturated solution (20.00 g in 70.00 mL of water) was added, and the mixture was shaken in a water bath at 37 °C for 30 min. Finally, the absorbance was determined at 760 nm using a Shimadzu UV-1800 spectrophotometer (Shimadzu Corporation; Japan). The results were expressed as milligram gallic acid equivalents.

#### 3.2.2. Condensed Tannins

Condensed tannins were evaluated in methanolic extracts with a concentration of 2.00 mg/mL, according to the method [35]. This extract was obtained by shaking 0.02 g of skins in 10 mL of methanol for 20 min and then centrifuging for 20 min at 4000 rpm. The supernatant was added (0.1 mL) into tubes that received 5.00 mL of a 1:1 solution prepared with vanillin 1% (w/v) in methanol and HCl 8% (v/v) in methanol. The tubes were then shaken in a water bath at 30 °C, and the absorbance was determined at 500 nm using a Shimadzu UV-1800 spectrophotometer (Shimadzu Corporation; Japan). The results were expressed as milligram catechin equivalents.

#### 3.2.3. Total Flavonoids

Total flavonoids were determined according to the method [36]. Peanut skin extracts were obtained with 70% ethanol at 20.00 mg/mL concentration. To obtain this extract, 1.00 g of peanut skins was immersed in 50 mL of 70% ethanol for 24 h at room temperature in the dark. The extract was then filtered and centrifuged at 700 rpm for 15 min. The supernatant was transferred to a volumetric flask, and the volume was made up to 50 mL; this was considered to be the final extract. Two tube series were prepared, and 0.5 mL of the extract was added to all of the tubes. In the first series, 4.3 mL of 80% ethanol was added to tubes that would receive an Al(NO_3_)_3_ solution. In the second series, 4.4 mL of 80% ethanol was added to the tubes that would not receive any Al(NO_3_)_3_. All the tubes then received 0.1 mL of a 1.00 M solution of CH_3_COOK. At this point, the first series of tubes received 0.1 mL of a 10% Al(NO_3_)_3_ solution. All the tubes were shaken for 40 min, and then absorbance was determined at 415 nm using a Shimadzu UV-1800 spectrophotometer (Shimadzu Corporation; Japan). The calculations took into account the differences in the readings between the treatments. The final results were expressed as milligram quercetin equivalents.

#### 3.2.4. DPPH Free Radical Scavenging Activity

Antioxidant activity was measured using the DPPH (2,2-diphenyl-1-picrylhydrazyl) radical assay [37] of ethanolic extracts with a 0.20 mg/mL concentration. The extracts were obtained from 0.01 g peanut skins and 50.00 mL of ethanol that were shaken for 15 min and centrifuged for 10 min at 2000 rpm. The supernatant (0.50 mL) was added to ethanol (3.0 mL) and a 60.00 μM DPPH solution. The absorbance was determined at 517 nm using a Shimadzu UV-1800 spectrophotometer (Shimadzu Corporation; Japan), after 45 min in the dark. DPPH free radical scavenging activity was calculated using the equation below:

$$\text{DPPH free radical scavenging activity }(\%)=[({\text{Abs}}_{\text{control}}-{\text{Abs}}_{\text{sample}})/({\text{Abs}}_{\text{control}})]\times 100$$

where Abs_control_ was the absorbance of DPPH radical + ethanol; Abs_sample_ is the absorbance of DPPH radical + peanut skin extract or trolox. The results were expressed as Trolox equivalent antioxidant capacity (TEAC).

#### 3.2.5. ABTS Free Radical Scavenging Activity

In this assay [38] the ABTS radical (2,2′-azino-bis(3-ethylbenzothiazoline-6-sulphonic acid)), which is generated by oxidation with potassium persulfate, was utilized in ethanolic extracts with a 0.40 mg/mL concentration. A stock solution of ABTS was prepared at 7.00 mM one day before the analysis, and the work solution was prepared by diluting the stock solution to 0.70 nm ± 0.02 nm at 734 nm absorbance. The extracts were obtained using the same method described for the DPPH assay with 0.02 g of peanut skins added into 50.00 mL of ethanol. The supernatant (20.00 μL) was added into a quartz cuvette along with the ABTS radical solution (2.00 mL), and the absorbance was determined at 734 nm for 6 min using a Shimadzu UV-1800 spectrophotometer (Shimadzu Corporation; Japan). ABTS free radical scavenging activity was calculated using the equation below:

$$\text{ABTS free radical scavenging activity }(\%)=[({\text{Abs}}_{\text{control}}-{\text{Abs}}_{\text{sample}})/({\text{Abs}}_{\text{control}})]\times 100$$

where Abs_control_ was the absorbance of ABTS radical + ethanol; Abs_sample_ is the absorbance of ABTS radical + peanut skin extract or trolox. The results were expressed as TEAC.

#### 3.2.6. Rancimat Oxidative Stability

The Oil Stability Index was evaluated according to Cd 12b-92 method [39] in Metrohm’s 743 Rancimat equipment (Metrohm Ltd, CH-9101 Herisau, Switzerland). Five grams of refined-bleached-deodorized (RBD) soybean oil free from synthetic antioxidants were added to 1 mL ethanolic peanut skins extracts and heated at 110 °C with an airflow of 9 L/h in the sample reaction tubes. To obtain the extracts used in this test, the yield of total phenolic content, as found in Section 2.2.1, was considered. To prepare each 100 mg/kg extract, 62.25 mg of peanut skins were immersed in 10.0 mL of ethanol or hexane for 24 h at room temperature in the dark. The extract was then filtered and centrifuged at 700 rpm for 15 min. The supernatant was transferred to a volumetric flask, and the volume was made up to 10.0 mL. The induction period was expressed in hours.

#### 3.2.7. Statistical Analysis

The experimental design was randomized with three replications. The results were analyzed (ANOVA) using the F-test. Mean comparisons were tested based on Tukey (*p* < 0.05) by SAS software. Correlation analyses were carried out with ASSISTAT 7.6 program.

## 4. Conclusions

Peanut skin samples contain bioactive compounds such as polyphenols, condensed tannins, and flavonoids. The antioxidant activity of peanut skins was confirmed using the DPPH and ABTS *in vitro* methods. The Oil Stability Index method confirmed that the ethanolic extracts retarded the oxidation process of soybean oil; besides DPPH and ABTS have shown increasing antioxidant activity by gamma radiation the Oil Stability Index method demonstrated that gamma radiation does not affect the antioxidant properties of the peanut skin ethanolic extracts when added in soybean oil. Total phenolic content, and antioxidant activity *in vitro*, are good indicators for measuring the antioxidant properties from plant extracts. However, tests in model system are indispensable in the assessment of the results of treatments such as gamma radiation.

## Figures and Tables

**Table 1:** Polyphenols of gamma-irradiated peanut skin extracts^1^.

	0.0 kGy	5.0 kGy	7.5 kGy	10.0 kGy
TPC 2 (mg GAE/g)	79.36 ± 1.94 c 3	80.73 ± 2.07 bc	88.31 ± 2.29 a	84.70 ± 1.32 ab
CT (mg CAT/g)	230.43 ± 4.65 a	203.71 ± 0.84 c	209.33 ± 2.12 c	221.43 ± 2.23 b
TFC (mg QUER/g)	4.37 ± 0.28 b	4.25 ± 0.17 b	5.01 ± 0.13 a	4.79 ± 0.24 ab

1 Data represent the mean ± standard deviation of each sample assayed in triplicate;

2 Abbreviations: TPC = total phenolic content, GAE = gallic acid equivalent, CT = condensed tannin, CAT = catechin, TFC = total flavonoid content, QUER = quercetin.

3 Means with different letters within a row indicate significant differences among doses by Tukey’s multiple test (*p* < 0.05).

**Table 2:** Induction period (h) 1 of soybean oil with added ethanolic peanut skin extracts or synthetic antioxidants.

	Control	TBHQ (200 mg/kg)	BHT (100 mg/kg)	Ethanolic extract from peanut skins (400 mg/kg)			
Dose (kGy)				0.0	5.0	7.5	10.0
Induction period (h)	5.72 ± 0.35 d 2	17.34 ± 0.13 a	6.47 ± 0.30 c	7.37 ± 0.03 b	7.22 ± 0.07 b	7.11 ± 0.19 b	7.30 ± 0.04 b

1 Data represent the mean ± standard deviation of each sample assayed in triplicate;

2 Means with different letters indicate significant differences (*p* < 0.05).
